# High-speed Fourier ptychographic microscopy based on programmable annular illuminations

**DOI:** 10.1038/s41598-018-25797-8

**Published:** 2018-05-16

**Authors:** Jiasong Sun, Chao Zuo, Jialin Zhang, Yao Fan, Qian Chen

**Affiliations:** 1 School of Electronic and Optical Engineering, Nanjing University of Science and Technology, No. 200 Xiaolingwei Street, Nanjing, Jiangsu Province 210094 China; 2 Jiangsu Key Laboratory of Spectral Imaging & Intelligent Sense, Nanjing, Jiangsu Province 210094 China; 3 Smart Computational Imaging Laboratory (SCILab), Nanjing University of Science and Technology, Nanjing, Jiangsu Province 210094 China

## Abstract

High-throughput quantitative phase imaging (QPI) is essential to cellular phenotypes characterization as it allows high-content cell analysis and avoids adverse effects of staining reagents on cellular viability and cell signaling. Among different approaches, Fourier ptychographic microscopy (FPM) is probably the most promising technique to realize high-throughput QPI by synthesizing a wide-field, high-resolution complex image from multiple angle-variably illuminated, low-resolution images. However, the large dataset requirement in conventional FPM significantly limits its imaging speed, resulting in low temporal throughput. Moreover, the underlying theoretical mechanism as well as optimum illumination scheme for high-accuracy phase imaging in FPM remains unclear. Herein, we report a high-speed FPM technique based on programmable annular illuminations (AIFPM). The optical-transfer-function (OTF) analysis of FPM reveals that the low-frequency phase information can only be correctly recovered if the LEDs are precisely located at the edge of the objective numerical aperture (*NA*) in the frequency space. By using only 4 low-resolution images corresponding to 4 tilted illuminations matching a 10×, 0.4 *NA* objective, we present the high-speed imaging results of *in vitro* Hela cells mitosis and apoptosis at a frame rate of 25 Hz with a full-pitch resolution of 655 *nm* at a wavelength of 525 *nm* (effective *NA* = 0.8) across a wide field-of-view (FOV) of 1.77 *mm*^2^, corresponding to a space–bandwidth–time product of 411 megapixels per second. Our work reveals an important capability of FPM towards high-speed high-throughput imaging of *in vitro* live cells, achieving video-rate QPI performance across a wide range of scales, both spatial and temporal.

## Introduction

High-throughput microscopy allows to high-content quantitative analysis of multiple events in a large population of cells, which is of crucial importance for many applications, such as personalized genomics, cancer diagnostics, and drug development^1,2^. Compared to fluorescence imaging, label-free quantitative phase imaging (QPI) approaches^3–5^ are particularly attractive due to their noninvasive and nontoxic properties. Various QPI techniques have been developed during the last decades, such as digital holography (DH)^6,7^, transport-of-intensity equation (TIE)^3^, and differential phase contrast (DPC)^8^ based methods, providing invaluable optical tools for biomedical research thanks to their unique capabilities to image optical thickness variation of living cells and tissues without the need for specific staining or exogenous contrast agents. Furthermore, due to the limited raw images (generally no more than 4 images) required for phase reconstruction, high-speed or even single-shot QPI has been demonstrated by employing fast switchable devices or using spatial/color multiplexing techniques^9–11^. However, the attainable space-bandwidth product (SBP) of these QPI techniques is fundamentally limited by the optical system used, resulting in a tradeoff between image resolution and field-of-view (FOV). While for high-throughput microscopy applications, it is always desirable to have a QPI technique that is able to record large FOV images without compromising the spatial and temporal resolution, allowing for simultaneous analysis of a large population of cells.

During the last decade, an enormous amount of research has been conducted to decouple imaging FOV and resolution from each other, creating high-resolution wide-field microscopic images based on novel computational techniques, such as synthetic aperture microscopy^12,13^, lens-free on-chip microscopy^14,15^, and Fourier ptychographic microscopy (FPM)^16^. FPM is a recently developed computational imaging technique that circumvents the SBP limit of a bright-field microscope by transforming the corresponding physical challenge into a mathematical optimization problem^16^. Rather than starting with high resolution and stitching together a larger FOV, FPM uses low numerical aperture (*NA*) objective lens to take advantage of its innate large FOV and stitches together images in Fourier space to recover high resolution. By sequentially illuminating the sample with different incident angles based on a programmable light-emitting diode (LED) array, different high spatial frequency components of the object spectrum can be shifted into the passband of the low *NA* objective. The underlying principle of resolution improvement in FPM is analogous to that of coherent aperture synthesis^17–20^ and structured-illumination^21–23^ imaging. But unlike synthetic aperture, FPM uses nonlinear optimization algorithms^16,24^ similar to translational diversity^25,26^ and ptychography^27–29^ to perform the reconstruction instead. A major advantage of FPM is that it can recover high-resolution wide-FOV complex images (including both amplitude and phase) with no moving parts. In addition, with digital wavefront correction strategy, FPM is able to achieve a much deeper depth of field (DOF) than that provided by a conventional high-magnification objective^30^, avoiding extra mechanical realignment^16^. Although significant progresses have been made in FPM for achieving larger SBP^31,32^, higher data acquisition efficiency^33–36^, and better reconstruction quality^36–41^ in the past few years, the underlying theoretical mechanism for high-accuracy phase imaging remains poorly understood, and high-speed high-accuracy QPI based on FPM is still an open quest. In order to improve the imaging speed, a source-coded FPM technique with a hybrid illumination scheme was proposed by Tian *et al*.^36^, which first captures four DPC images to cover the bright-field LEDs, and then uses random multiplexing with eight LEDs to fill in the remaining dark-field Fourier space region. By reducing the number of acquired images to 21, large-SBP imaging of live cells *in vitro* with a speed of 1.25 Hz was demonstrated. However, the imaging speed is still insufficient for many high-speed QPI applications, where a frame rate about 25 Hz is typically needed for video-rate imaging.

To this end, here we report a high-speed FPM technique based on programmable annular illuminations. It should be noted that the main obstacle for FPM to achieve high-speed QPI is compounded by three factors: unstable phase retrieval, large image datasets, and long acquisition time. Firstly, in order to achieve high-accuracy phase recovery for unstained samples using FPM, the optical transfer function (OTF) of FPM is derived and analyzed. It has been found that in DPC, the low-frequency phase information is usually transferred poorly and can hardly retrieved correctly^42^. The same applies to FPM, where the phase contrast provided by the asymmetric illumination also results in uneven sensitivity to phase at different spatial frequencies. Based on the derived absorption transfer function (ATF) and phase transfer function (PTF) of FPM, we found that the low-frequency phase information results only from illumination angles (*NA*_*ill*_) matching the objective numerical aperture (*NA*_*obj*_), which is very different from the situation of amplitude reconstruction. This means that the low-spatial-frequency phase information can only be correctly recovered if the LEDs are precisely located at the edge of the objective *NA* in the frequency space. Thus, in order to guarantee accurate phase reconstruction, the LED array’s position need precise adjustment. Secondly, according to the PTF and the data redundancy requirement of FPM, we propose a new illuminating strategy to reconstruct a large SBP with very few images. Our new method, termed AIFPM, uses an annular illumination scheme: by only lighting up LED elements located on a ring with the illumination *NA* matching the *NA*_*obj*_, only 4–12 bright-field raw images are required to achieve high-accuracy phase retrieval with significantly reduced data redundancy (3–20 times). Thirdly, in conventional FPM, one needs to capture many dark-field images with very low intensity, which requires longer exposure time (typically ≫ 30 ms) in order to maintain a reasonable signal-to-noise ratio (SNR). In contrast, AIFPM only uses bright-field images to expand the effective *NA* to the incoherent diffraction limit, while reducing the exposure time for each image to only 10 ms. Thus, the total data acquisition time is only 0.12 s for 12-LED scanning, and can even be reduced to 0.04 s if the fastest 4-LED scanning mode is used. This speed is finally suitable for the video-rate (25 Hz) *in vitro* QPI applications (*e.g*., cell division processes), enabling capture of fast subcellular dynamics (*e.g*., vesicle tracking) without introducing motion blur.

Based on AIFPM, we demonstrate high-speed large-SBP imaging results for both growing and confluent samples *in vitro*. By using only 4 annular illuminations per reconstruction, we achieve a space–bandwidth–time product (SBP-T) of 411 megapixels per second (1.77 *mm*^2^ FOV, 328 *nm* half-pitch resolution, and 16.45 megapixels captured in 0.04 s), nearly approaching the theoretical limit of the camera’s data transfer rate (419 megapixels per second, 4.19 megapixels captured in 0.01 s). Due to this large SBP-T, we observe fast cell dynamics both on the sub-cellular level and across the entire cell population, revealing an important capability of FPM towards high-speed high-throughput imaging of *in vitro* live cells.

## Materials and Methods

### Optical setup

**Figure 1:**
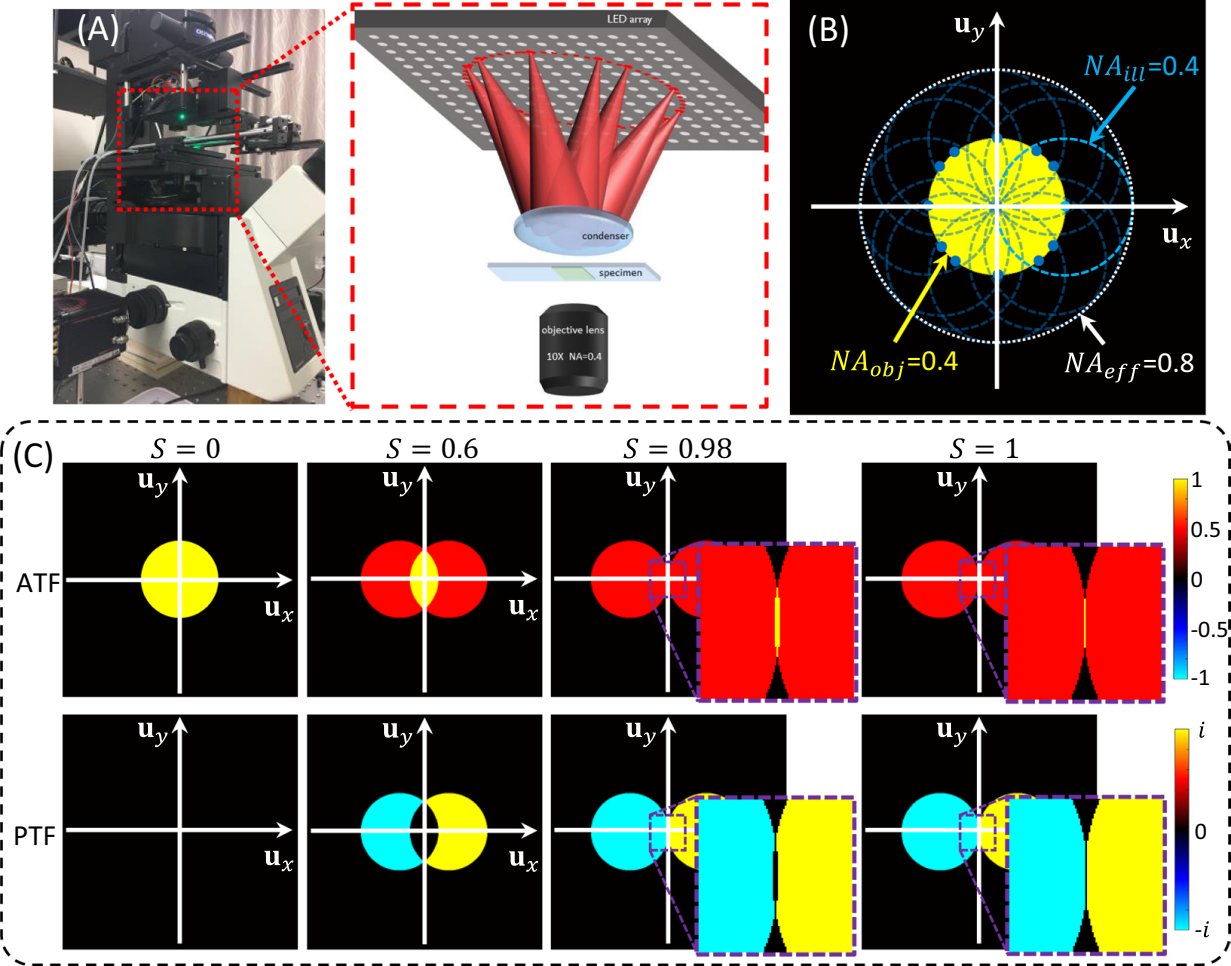
Optical setup of the annular illuminating LED array and condenser-based AIFPM platform. (**A**) The experimental setup involves an LED array board, a cemented doublet condenser, an Olympus IX73 microscope with an Olympus UPlanSApo 10× (0.40 *NA*) objective lens, and a scientific CMOS camera. (**B**) Fourier coverage using AIFPM (12 images), with an acquisition time of 0.12 s per frame. (**C**) OTFs under different illumination angles for FPM.

As depicted in Fig. 1, the AIFPM setup in this paper consists of three major components: a programmable LED array, a condenser lens, and a microscopy imaging system. The commercial, multi-wavelength surface-mounted LED array (4 *mm* spacing) is placed at the front focal plane of the condenser (a cemented doublet with the focal length of 50 *mm*). The central wavelength of green channel is 525 *nm*, and the spectral linewidth is ∼20 *nm*. During the imaging process, the LED elements located on a ring, which has a radius of 20 *mm*, are lighted up sequentially with the same *NA*_*ill*_ of 0.4 from different illumination angles. Considering the 4 *mm* spacing between two neighboring LEDs and the ring’s radius of 20 *mm*, there are 12 LED elements on the board which can provide annular illuminations [whose spatial coordinates are (0 *mm*, ±20 *mm*), (±20 *mm*, 0 *mm*), (±12 *mm*, ±16 *mm*), (±16 *mm*, ±12 *mm*)], as shown in Fig. 1(B). Since the accuracy of the illumination angle is a vital point in AIFPM, the LED array is fixed on a combined three axis translation stage to adjust its position mechanically. In order to improve the measuring speed and decrease the exposure time, all the LED elements are driven statically with enhanced output current using a self-made LED controller board with an FPGA controller. The imaging system consists of two parts: a commercial bright-field microscope (IX73, Olympus, Japan) including a 10×, 0.4 *NA* objective (UPLSAPO10×, Olympus), and a scientific CMOS camera (Hamamatsu ORCA-Flash 4.0 C13440, 6.5 *μm* pixel pitch). The camera is synchronized with the LED array by the same controller via two coaxial cables that provide the trigger and monitor the exposure status. The data are transferred to the computer via a CameraLink interface. We experimentally measure the system frame rate to be 100 Hz for capturing full-frame (2048 × 2048) 16-bit images. Thus, in the fastest AIFPM model, those 4 images [whose corresponding LEDs’ spatial coordinates are (−12 *mm*, ±16 *mm*), (16 *mm*, ±12 *mm*)] are captured within 0.04 seconds (10 ms exposure time per image), corresponding to a phase imaging frame rate of 25 Hz. Since all the raw images acquired in AIFPM are captured in bright-field illumination, 10 ms exposure time is enough for the 16-bit scientific CMOS camera to produce high-quality images.

### Optical transfer function for FPM

Although FPM has found many successful applications in high-throughput screening and digital pathology due to its effective expansion in SBP, the underlying theoretical mechanism of the QPI as well as the phase retrieval accuracy has not been clearly revealed till now. Different from TIE^3,43–45^ and DPC^8,42,46,47^ based phase retrieval methods in which the phase reconstruction process may boil down to two dimensional (2D) deconvolutions, FPM reconstructs the complex amplitude distribution of the sample in an iterative manner, thus its OTF is rarely studied. Here, in order to quantify how phase information is converted into intensity and reveal the phase measuring accuracy for FPM, we give a derivation of the ATF and PTF of FPM.

Consider a weak object with complex transmission function

1 $$\begin{array}{rcl}t({\bf{x}}) & = & \sqrt{{I}_{obj}({\bf{x}})}{e}^{i{\varphi }({\bf{x}})}=\sqrt{{e}^{a({\bf{x}})}}{e}^{i{\varphi }({\bf{x}})}={e}^{\frac{1}{2}a({\bf{x}})+i{\varphi }({\bf{x}})}\\  & \approx  & 1+\frac{1}{2}a({\bf{x}})+i{\varphi }({\bf{x}})\end{array}$$

where **x** is the 2D spatial coordinate in the real space, *t*(**x**) is the complex amplitude of the object, *I*_*obj*_(**x**) and *ϕ*(**x**) present its intensity and phase distributions. For simplification, we define the absorption distribution of the object as *I*_*obj*_(**x**) = *e*^*a*(**x**)^ and adopt a weak object approximation^48,49^. In order to analyse the transfer function, we take the Fourier transform on the both sides of Eq. 1 and obtain the Fourier spectrum of *t*(**x**)

2 $$\begin{array}{l}T({\bf{u}})=\delta ({\bf{u}})+\frac{1}{2}A({\bf{u}})+i{\rm{\Phi }}({\bf{u}})\end{array}$$

where **u** is the corresponding 2D coordinate in the Fourier space, *δ*(**u**) is a Dirac Delta function, *A*(**u**) and Φ(**u**) present the spectrum of absorption and phase distributions. Once the object is illuminated with a tilted coherent plane wave in FPM, its spectrum will be shifted in the Fourier space^16^. Thus, with the tilted illumination angle **u**_0_, the Fourier spectrum of the transmitted complex wave-front is

3 $$\begin{array}{rcl}{W}_{obj}({\bf{u}}) & = & T({\bf{u}}-{{\bf{u}}}_{0})\\  & = & \delta ({\bf{u}}-{{\bf{u}}}_{0})+\frac{1}{2}A({\bf{u}}-{{\bf{u}}}_{0})+i{\rm{\Phi }}({\bf{u}}-{{\bf{u}}}_{0})\end{array}$$

Before reaching the digital camera, the complex wave-front is low-pass filtered by the pupil function in the Fourier domain

4 $$\begin{array}{l}{W}_{cam}({\bf{u}})={W}_{obj}({\bf{u}})P({\bf{u}})\end{array}$$

here *P*(**u**) presents the pupil function of the objective lens. Assuming that *P*(**u**) is an ideal low pass filter with the cut-off frequency of $\frac{N{A}_{obj}}{\lambda }$, the complex wave-front spectrum at the camera plane can be written as

5 $$\begin{array}{rcl}{W}_{cam}({\bf{u}}) & = & \{\begin{array}{cc}\delta ({\bf{u}}-{{\bf{u}}}_{0})+\frac{1}{2}A({\bf{u}}-{{\bf{u}}}_{0})P({\bf{u}})+i{\rm{\Phi }}({\bf{u}}-{{\bf{u}}}_{0})P({\bf{u}}), & |{{\bf{u}}}_{0}|\le \frac{N{A}_{obj}}{\lambda }\\ \frac{1}{2}A({\bf{u}}-{{\bf{u}}}_{0})P({\bf{u}})+i{\rm{\Phi }}({\bf{u}}-{{\bf{u}}}_{0})P({\bf{u}}),else & \end{array}\end{array}$$

where $|{{\bf{u}}}_{0}|\le \frac{N{A}_{obj}}{\lambda }$ denotes the bright-field imaging condition when illumination *NA* is not larger than objective *NA*. At last, by adopting convolution process between *W*_*cam*_(**u**) and its complex conjugate term *W*′_*cam*_(**u**), we can get the intensity spectrum for bright-field imaging in FPM as

6 $$\begin{array}{rcl}{I}_{cam}({\bf{u}}) & = & {W}_{cam}({\bf{u}})\ast {W^{\prime} }_{cam}({\bf{u}})\\  & \approx  & \delta ({\bf{u}})+\frac{1}{2}A({\bf{u}})[P({\bf{u}}+{{\bf{u}}}_{0})+P({\bf{u}}-{{\bf{u}}}_{0})]+i{\rm{\Phi }}({\bf{u}})[P({\bf{u}}+{{\bf{u}}}_{0})-P({\bf{u}}-{{\bf{u}}}_{0})]\end{array}$$

here we neglect the high order convolution terms between *A*(*u*) and Φ(*u*) to linearize the problem^48,49^. Note that this bright-field imaging function of tilted coherent illumination also can be derived from the 4D partially coherent transfer function or the transmission cross coefficient (TCC) model^50,51^. In Eq. 6, the intensity spectrum can be separated into three terms: background term, absorption transfer term, and phase transfer term. Thus, the ATF and PTF for bright-field imaging of a weak object in FPM can be represented as

7 $$\begin{array}{rcl}ATF({\bf{u}}) & = & \frac{1}{2}[P({\bf{u}}+{{\bf{u}}}_{0})+P({\bf{u}}-{{\bf{u}}}_{0})]\\ PTF({\bf{u}}) & = & i[P({\bf{u}}+{{\bf{u}}}_{0})-P({\bf{u}}-{{\bf{u}}}_{0})]\end{array}$$

In order to analyze the relationship between OTF and the illumination *NA*, we define a normalized factor $S=\frac{N{A}_{ill}}{N{A}_{obj}}$ (so-called coherent parameter) to represent the normalized illumination angle from each single LED for different objective lens. When *S* ≤ 1, camera will capture bright-field images, otherwise dark-field images will be recorded. As shown in Fig. 1(C), the support areas of ATF and PTF for bright-field imaging in FPM are decided by the normalized illumination angle *S*. Notably, as long as *S* ≤ 1, the low frequency area close to zero frequency in the Fourier space is covered under different ATFs, which means these low frequency intensity components are effectively transferred and recorded by the camera. However, considering the PTF of FPM, on-axis illumination (*S* = 0) provides only the intensity without any phase information. This is the reason why pure-phase object can hardly be observed under normal bright-field illumination, such as unstained live cells. In addition, axial symmetrical illuminations also cannot transfer phase information into intensity contrast because two anti-symmetrical (positive and negative) components of PTFs just cancel each other out. More importantly, for low-frequency phase components (near the origin), they can be completely covered only if *S* = 1, as presented in Fig. 1(C). In other words, the low frequency phase information is very difficult to be recorded in the raw images of FPM because it can only be transferred into intensity by matched tilted illuminations (the LED is precisely located at the edge of the *NA*_*obj*_). This is why high accuracy phase reconstruction is much more difficult to achieve for FPM comparing to intensity recovery.

### FPM based on annular illuminations

In order to achieve high accuracy phase retrieval using FPM, the maximum illumination *NA* of bright-field images should be equal to the objective *NA* (*S*_*max*_ = 1) according to the OTF of FPM. Furthermore, as shown in Fig. 1(C), when the illumination angle matches the *NA*_*obj*_, the frequency support areas of the ATF and PTF can be maximized with a synthetic *NA* reaching two times of the *NA*_*obj*_ along the illumination direction. In other words, those particular bright-field raw images contain the maximum amount of information, especially the valuable low frequency phase information, compared with other bright-field or dark-field images. Therefore, using matched annular illumination not only improves the recovery accuracy, but also reduces the number of required raw images. According to the observation and analysis above, we propose a video-rate high-throughput phase retrieval method based on FPM, named AIFPM. In AIFPM, we only light up a few LED elements on a ring to provide matched tilted illuminations (*NA*_*ill*_ = *NA*_*obj*_) from different angles, as presented in Fig. 1(B). In this case, the number of required raw images can be limited between 4 and 12, expanding the resolution beyond coherent diffraction limit without sacrificing recovery accuracy.

**Figure 2:**
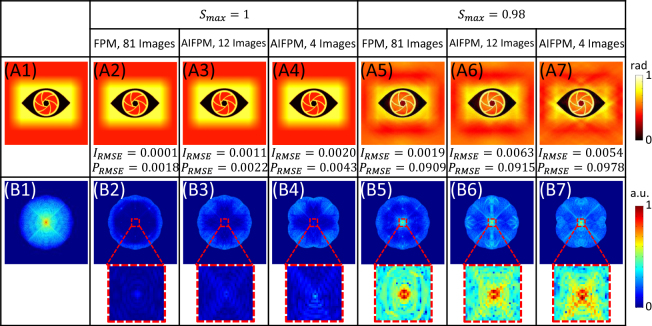
Simulation results of the recovered phase maps under different situations. (**A1–B1**) The phase image and its Fourier spectrum of the simulated pure-phase object. (**A2–A7**) Recovered phase maps using ordinary FPM and AIFPM under different illumination conditions. (**B2–B7**) The spectrums of the phase difference between (**A2–A7**) and (**A1**).

Aiming to demonstrate the theoretical effectiveness of AIFPM, Fig. 2 presents the simulation results of the recovered phase maps with different amounts of raw data and different maximum normalized illumination angles *S*_*max*_. The simulation parameters were chosen to realistically model our FPM experimental system, and Fig. 2(A1–B1) display the phase image and its Fourier spectrum of a pure-phase object. Instead of simulating a complex amplitude object, here we only investigate pure-phase object and focus on the phase retrieval accuracy since the unstained live cells generally have very little intensity contrast and their intensity value can be regarded as a constant. Next, we implement three different FPM schemes. One is the ordinary FPM with 81 bright-field images (all those 81 bright-field LEDs are lighted up sequentially). The other two are AIFPM using fewer number of particular bright-field images respectively (12 images and 4 images). When the maximum normalized illumination angle *S*_*max*_ = 1, once the data redundancy requirement for FPM is satisfied, high accuracy phase recovery can be achieved, as shown in Fig. 2(A2–A4). In addition, the spectrum of the phase difference between Fig. 2(A1) and (A2-A4) are shown in Fig. 2(B2–B4) with their enlarged low frequency regions. It can also be seen that even the AIFPM only uses 4 images, the low frequency phase components can still be retrieved accurately, despite a slightly increased root-mean-square error (RMSE). This demonstrates that AIFPM can significantly improve the measuring speed without sacrificing recovery accuracy. It may be doubted that the overlapping ratio of the 4-image scheme is generally insufficient for accurate FPM reconstruction (at least 35%)^40^. However, if the sample is a pure phase object with weak phase variations, the data redundancy requirement can be further reduced by half because one image can be used to update two aperture regions according to its PTF [see Fig. 1(c)]. Thus, an extra uniform intensity constraint is applied to the full spectrum at the end of each iteration so that AIFPM can use only 4 images to recover the phase information within totally 8 frequency apertures [Fig. 2(A4,B4)]. On the other hand, when the actual maximum normalized illumination angle *S*_*max*_ = 0.98 < 1, the recovered phase distributions will be distorted significantly for both ordinary FPM and AIFPM, as shown in Fig. 2(A5–A7). Note that for the plane wave incident along the optical axis (*S* = 0), the ATF can cover all the low frequency intensity components, but the PTF contains no phase information. Only if the maximum illumination *NA* equals *NA*_*obj*_, the low frequency phase components can be reconstructed accurately. Therefore, when the actual maximum normalized illumination angle *S*_*max*_ < 1, no matter how large the data redundancy is used in FPM, high accuracy phase recovery can never be achieved. This reveals the major difficulty for FPM in high quality phase retrieval. Thus, in the AIFPM sytem, we utilize a combined three-axis translation stage to adjust LED array’s position carefully in order to guarantee that the condition *NA*_*ill*_ = *NA*_*obj*_ can be precisely satisfied.

### Comparison of AIFPM and DPC

**Figure 3:**
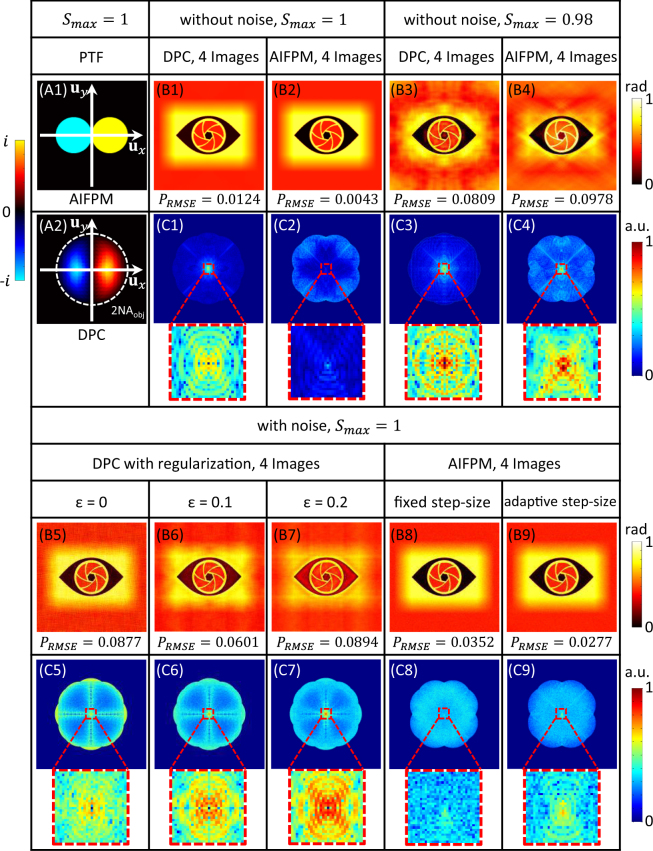
Comparison of AIFPM and DPC under different simulation conditions. (**A1–A2**) PTFs of AIFPM and DPC. (**B1–B4**) Recovered phase maps using AIFPM and DPC under different illumination schemes without noise. (**B5–B9**) Recovered phase maps using DPC and AIFPM with different regularization parameters and updating step-sizes while adding same Gaussian white noise in their raw images. (**C1–C9**) The spectrums of the phase difference between (**B1–B9**) and the ideal input.

Compared with DPC based phase retrieval techniques, AIFPM also only uses 4 raw images to perform high accuracy phase recovery as demonstrated above. However, there are three essential differences between them. Firstly, the DPC is not an iterative phase recovery method, so it is faster and easier to implement than AIFPM. Secondly, as DPC can be viewed as a one-step deconvolution algorithm in the frequency domain, the pixel resolution of the raw intensity image captured should be at least higher than the Nyquist sampling resolution imposed by 2 *NA*_*obj*_ [Fig. 3(A2)]. Thus, for most DPC-based phase retrieval systems, a 2× camera adaptor or a digital camera with smaller pixel-size is usually required to satisfy the Nyquist sampling theorem. However, considering FPM as an iterative super-resolution algorithm, its required pixel resolution of the raw images is just determined by the coherent diffraction limit (*NA*_*obj*_). In other words, for a same microscopic system, AIFPM can achieve higher pixel resolution without aliasing or sacrificing FOV comparing with DPC. Thirdly, the response of DPC’s PTF is close to zero near the origin and cut-off frequency 2 *NA*_*obj*_, while the response of AIFPM’s PTF remains close to unity over all the frequency support region, as shown in Fig. 3(A1,A2). Since DPC is an inverse filtering algorithm and extremely small values exist in its PTF, regularization strategy is always needed to prevent the noise amplification due to the ill-conditioned nature of the inverse problem. With the increase of noise level, the selected regularization parameter *ε* should increase correspondingly, which often leads to low frequency regularization error in phase reconstruction.

In Fig. 3(B5–B7), we present a set of simulation results with different regularization parameters using DPC. These three phase recovery results are obtained from the same set of raw data which are corrupted by additive white Gaussian noise with standard deviation of 0.015 when *S*_*max*_ = 1. In order to suppress the influence of the imaging noise, three different regularization parameters *ε* are used (0, 0.1, 0.2). The corresponding phase RMSEs are also calculated and displayed below each figure. In addition, the Fourier spectrums of the phase recovery errors are shown in Fig. 3(C5–C7), respectively. As can be seen, a smaller regularization parameter is helpful to recover the low frequency phase information more completely, but at the expense of insufficient noise suppression. On the other hand, increasing the regularization parameter can reduce the high frequency error, but the low frequency phase component cannot be recovered accurately. Therefore, finding a proper regularization parameter *ε* is very important for DPC to achieve reliable phase reconstructions under different noise conditions. In contrast, since the absolute values in AIFPM’s PTF remain close to one over all the frequency support region, there is no need for regularization. Based on AIFPM, phase recovery results and their corresponding phase error spectrums are shown in Fig. 3(B8,B9) and (C8,C9), respectively. After 20 iterations with a fixed step-size 0.5, the phase RMSE obtained by AIFPM (0.0342 rad) is even smaller than the minimum phase RMSE obtained by using DPC (0.0539 rad). If we further implement an adaptive step-size strategy for AIFPM reconstruction, the phase retrieval quality can be further improved without any regularization process (RMSE reduces to 0.0252 rad). These simulation results demonstrate that AIFPM is more suitable than DPC for dealing with different noisy conditions. Note that this adaptive step-size updating strategy is also used in the experiments for achieving high-quality phase retrieval of living cells.

Last but not least, besides these three differences between AIFPM and DPC, there is one common precondition for both of them to achieve high-accuracy phase retrieval: the maximum normalized illumination angle *S*_*max*_ = 1. It has been demonstrated that the maximum normalized illumination angle is the key point in AIFPM for achieving high accuracy phase recovery shown above. However, it is rarely known that it is also a major pre-requisite in DPC. As displayed in Fig. 3(B1,B2), when the maximum normalized illumination angle *S*_*max*_ = 1, high accuracy phase recovery can be achieved with both AIFPM and DPC. However, when the LED array’s height is misaligned resulting in *S*_*max*_ = 0.98, the DPC cannot give the correct phase reconstruction result as well, as presented in Fig. 3(B3,B4). Thus, no matter which method is used, AIFPM or DPC, *S*_*max*_ = 1 should be guaranteed.

### Sample preparation

The plano-convex microlens array (MLA150-5C, Thorlabs), with a pitch of 150 *μm* (square), a lens diameter of 146 *μm*, a radius of 2380 *mm*, and a focal length of 5.2 *mm*, is located upside down on a cover glass with filled water between them to satisfy the weak phase object approximation. HeLa cells were seeded (at an initial density of 300 cells/*cm*^2^) in a 35 *mm* glass-bottom Petri dish in Dulbecco’s Modified Eagle’s Medium (DMEM) supplemented with 10% FBS, and 1% penicillin streptomycin. Cells were incubated at 37 °C in humidified atmosphere of 5% carbon dioxide for 8 hours to allow attachment. After that, cells were washed twice with PBS and pre-warmed fresh medium was added. Then, the cells were placed in the 37 °C incubator of the microscope with 5% *CO*_2_ for long-term time-lapse imaging.

### Computation platform used for AIFPM

Our reconstructions are performed using MATLAB (Version R2015b, MathWorks, Natick, Massachusetts) on a laptop computer equipped with a 2.60 GHz central processing unit (Intel Core i5-3320M) and 8 GB of random-access memory. In the reconstruction, we divided each full-FOV raw image (2048 × 2048 pixels) into 10 × 10 sub-regions (256 × 256 pixels each), with a 60-pixel overlap on each side of neighboring sub-regions. Each set of images was then processed by the algorithm described above to create a high-resolution reconstruction having both intensity and phase (768 × 768 pixels). Finally, all high-resolution reconstructions were combined using the alpha-blending stitching method to create the full-FOV high-resolution reconstruction. For each 256 × 256 pixels sub-region with an upsampling factor of 3, the processing time of 20 rounds iterative recovery routine takes ∼10 s. The total processing time for the full FOV was nearly 20 minutes, which could be further reduced by implementing GPU acceleration rather than MATLAB.

## Results

### Characterization of a microlens array

**Figure 4:**
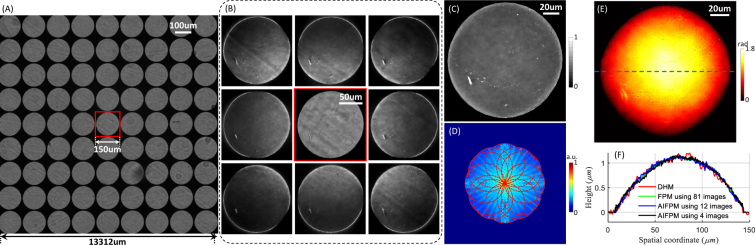
Characterization of a microlens array to evaluate the phase recovery accuracy of AIFPM. (**A**) The full FOV of one raw low resolution image. (**B**) The enlarged red-boxed region of one microlens in the middle of the FOV and 8 raw images for the same microlens corresponding to 8 different illumination directions with the same *NA*_*ill*_ = 0.4. (**C–E**) The intensity, Fourier spectrum, and quantitative phase image reconstructed using AIFPM with 12 images. (**F**) The thickness profiles for the microlens taken along the blue dashed line in (**E**) using digital holography, ordinary FPM, and AIFPM.

To demonstrate the accuracy of the phase reconstruction for AIFPM, a plano-convex microlens array (MLA150-5C, Thorlabs) was measured with the AIFPM system. This microlens array is specifically designed for Shack-Hartmann sensor applications, with a pitch of 150 *μm* (square), a lens diameter of 146 *μm*, a radius of 2380 *mm*, and a focal length of 5.2 *mm*. In the experiment, this microlens array was placed upside down on a cover glass and immersed in water so that it can be regarded as a weak phase object. In order to compare the phase retrieval accuracy of conventional FPM and AIFPM, we captured all the 81 bright-field images with the LED array’s position finely adjusted (*S*_*max*_ = 1). Figure 4(A) shows the raw low resolution image corresponding to *S* = 0. One microlens in the center of the FOV is enlarged and shown in Fig. 4(B), outlined with a red box. The other 8 images in Fig. 4(B) are the enlarged regions for the same microlens corresponding to 8 different illumination directions with the same normalized illumination angle *S* = 1. It can be seen that when the normalized illumination angle *S* = 1, the feature of the recorded raw images in FPM is quite similar to that of the DPC. Figure 4(C–E) respectively show the intensity, Fourier spectrum, and quantitative phase image reconstructed by AIFPM using 12 annular illumination images. After converting the phase value to the real thickness [the refractive index of lens material (fused silica) is 1.46 at 525 *nm*], the thickness profile for this microlens taken along the blue dashed line in Fig. 4(E) is shown in Fig. 4(F). Meanwhile, the thickness profiles for the same microlens recovered by using ordinary FPM with 81 raw images and AIFPM with 4 images are also compared quantitatively in Fig. 4(F), showing a reasonable agreement. To assess the accuracy of the phase measurement, the same sample was also measured using a digital holographic microscope (DHM) system equipped with a 60×, 0.85 *NA* microscope objective (laser wavelength 632.8 *nm*). After phase unwarping and converting to the physical thickness [the refractive index of lens material (fused silica) is 1.457 at 632.8 *nm*], the line profile of one lens on the same microlens array is shown in Fig. 4(F). Although this line profile surfers from coherent noise, its corresponding curvature radius after arch fitting is about 2398 *mm*, presenting an acceptable agreement with the nominal value 2380 *mm*, as same as the results obtained by AIFPM with only 12 or 4 images. These results demonstrate that AIFPM has great potential to increase FPM measuring speed without compromising the phase recovery accuracy.

### Experimental comparison of AIFPM and DPC

**Figure 5:**
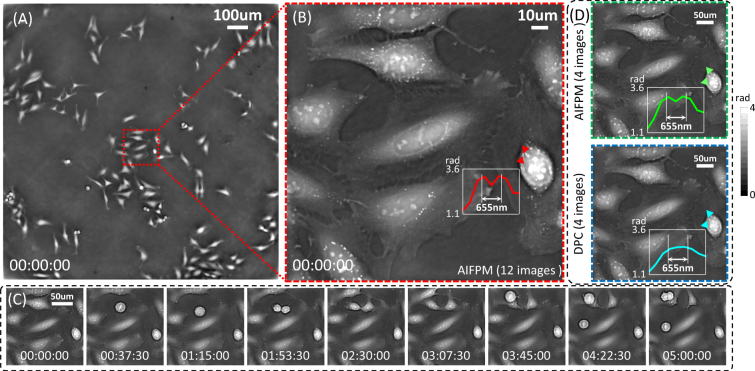
Time-lapse large-SBP phase reconstruction of unstained HeLa cells undergoing division using AIFPM and DPC. (**A**) One frame of the phase reconstruction with a full FOV of 1.77 *mm*^2^. (**B**) Enlarged image of the red-boxed region in (**A**). (**C**) Several frames of reconstructed video (see Supplementary Video 1) from a zoom-in of the selected small area. (**D**) Comparison of phase reconstructions obtained by using DPC and AIFPM with 4 images.

Figure 5 shows a few frames from a time-lapse video of the human cervical adenocarcinoma epithelial (HeLa) cell division process over the course of 5 h at a frame rate of 8.33 Hz, as well as a comparison of DPC and AIFPM using different raw images. These data were captured using our AIFPM system (12 images per phase reconstruction, 10 ms exposure time per image). By utilizing AIFPM with 12 images, one frame of the full-FOV phase reconstruction is shown in Fig. 5(A) and a few frames of the video for a single zoom-in are shown in Fig. 5(B,C). To evaluate the resolution of our method, a line profile of subcellular feature is presented in Fig. 5(B) and it is shown that our AIFPM with 0.8 synthetic *NA* is able to achieve 655 full-pitch resolution at a wavelength of 525 *nm* across a wide FOV of 1.77 *mm*^2^. In this enlarged region, cells were undergoing mitosis and dividing into two daughter cells (see Supplementary Video 1 for the corresponding full 5-hour time-lapse QPI movie). Furthermore, in order to compare the performance of AIFPM and DPC, we also provide the phase recovery results using DPC technique. Those 4 raw images of DPC were generated by summing up half of the 12 images to simulate 4 different annular tilted illuminations for DPC reconstruction. In addition, those 4 low resolution raw images for DPC were enlarged three times with bilinear interpolation before deconvolution in order to generate a final phase reconstruction with the same image size as the one obtained by AIFPM. It should be noted that since the interpolation does not introduce any additional information content of the raw image, it cannot restore frequencies distorted by aliasing. Thus, for our current experimental system, the theoretical imaging resolution of DPC is fundamentally limited by the camera’s pixel-size, instead of 2*NA*_*obj*_. By using 4-image AIFPM and DPC methods, two phase recovery results of the same selected region at the same time point are displayed in Fig. 5(D). It can be seen that, the low frequency phase components in Fig. 5(D) are well recovered using both AIFPM and DPC. This reveals that AIFPM is able to obtain high accuracy phase recovery results comparable to DPC once the LED array’s position is finely adjusted (*S*_*max*_ = 1). However, comparing the line profiles of the same subcellular feature in Fig. 5(D), the resolution of DPC is compromised and limited by the camera’s pixel-size (Nyquist sampling resolution 1.5 *μ*m), while the AIFPM effectively surpasses this limit and resolves the two closely spaced features with distance of 655 nm. These results suggest that the AIFPM successfully relaxes the pixel resolution requirement in DPC and can provide high-accuracy aliasing-free phase reconstruction with only 4 images.

### Video-rate phase imaging of HeLa cells *in vitro* using AIFPM

**Figure 6:**
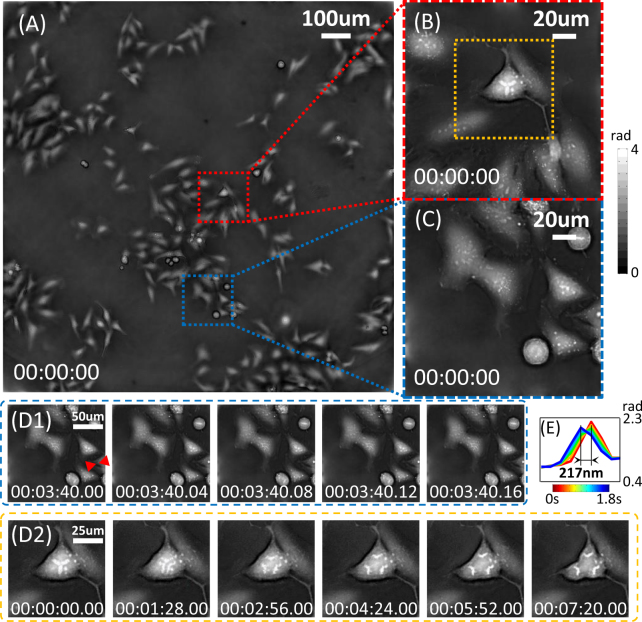
Video-rate phase imaging of HeLa cells *in vitro* using AIFPM with 4 raw images and an acquisition time of 0.04 s per frame. (**A**) One frame of the full FOV phase reconstruction using a 10× objective and achieving 0.8 *NA* resolution. (**B,C**) Enlarged images of the red-boxed and blue-boxed regions in (**A**). (**D1,D2**) Sample frames of reconstructed video (see Supplementary Video 2) for zoom-in view of two selected regions respectively at the maximum frame rate of 25 Hz within 1 s or at about 1.47 min intervals across 7.33 min. (**E**) Line profiles for tracking subcellular features’ sub-pixel movement within 1.8 s.

AIFPM also can be used to observe samples across short time scales (about 7 min) with video-rate acquisition speeds (25 Hz). An example frame from a reconstructed large-SBP phase video of HeLa cells is shown in Fig. 6(A). We use AIFPM to achieve the same SBP as ordinary FPM (0.8 *NA* resolution across a 10× FOV), but with only 4 images instead of more than 12 bright-field images. As a result, we significantly decrease the capture time to 0.04 s per round with 10 ms exposure time. Two selected zoom-in regions are shown in Fig. 6(B,C) and a few frames of the video for these two regions at different time scale are shown in Fig. 6(D1,D2). In the two zoom-ins of the phase images shown in Fig. 6(B,C), subcellular features, such as cytoplasmic vesicles and pseudopodium, are clearly observed. In the fast time scale, plasmids migration and other organelle motions were observed in a short time scale and at a small length scale in Fig. 6(D1). By extracting centroid coordinates or line profiles in Fig. 6(D1), subcellular features and their sub-pixel movement can be tracked precisely over time [Fig. 6(E)]. Furthermore, as shown in Fig. 6(D2), a unique mitotic event of the tri-daughter division can be observed in the middle time scale across 7 mins. In the beginning, the spindle was formed completely but the mitotic planes appear to be highly abnormal in contrast to normal bi-polar divisions, forming star-shaped aggregates with three centrosome pole regions. And during anaphase [Fig. 6(D2)], chromosome segregation moved toward three poles from the center under the traction of the spindle, leading to three daughter cells. Since each high resolution phase image was recorded in 0.04 s, all these retracting, extending, reorganizing, migrating, and maturing processes were recovered accurately avoiding motion blur, which usually occur in ordinary FPM for its large number of raw images.

### Long-term time-lapse multi-modal imaging of HeLa cell division in culture

**Figure 7:**
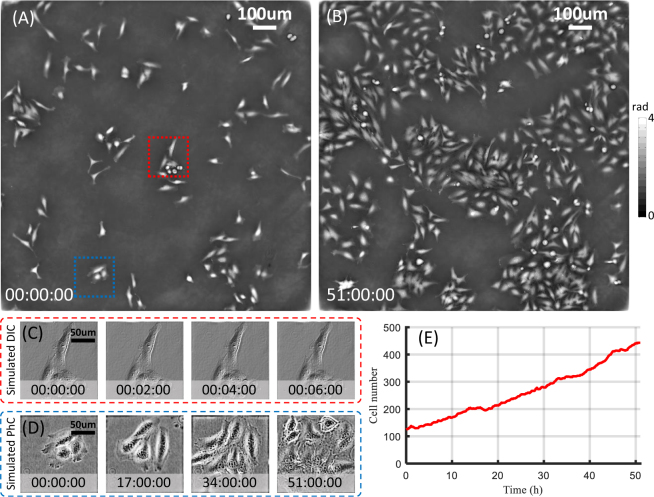
Long-term time-lapse multi-modal imaging of HeLa cells dividing in culture at 2 minutes intervals across 51 hours. (**A,B**) Two frames of the full FOV phase reconstruction at the beginning and the end of the measurement procedure. (**C,D**) Simulated PhC and DIC images for zoom-in view of two selected regions in (**A**) at different time points (see Supplementary Video 3). (**E**) The change of cell number over the culture passage period.

Thanks to the noninvasive and nontoxic properties of FPM, AIFPM can also be served as a multi-modal imaging tool for the visualization and quantity analysis of morphology and statistics variation of HeLa cells over a long-term period of time. Figure 7(A,B) show two frames, seperated in time by 51 h, from a long-term time-lapse movie of the HeLa cell division process over the period of 51 h, created with one phase image per two minutes reconstructed from 12 raw images using our AIFPM system (see Supplementary Video 3). In order to study the cells’ morphology and provide more useful images for biologists who are used to observe living cell samples by visual inspection, we further selected two different partial regions in the full FOV and create the simulated phase contrast (PhC) and differential interference contrast (DIC) images [Fig. 7(C,D)] from quantitative phase maps [corresponding to the red-boxed region and blue-boxed region in Fig. 7(A) respectively]. Different from quantitative phase images, PhC and DIC images are particular valuable since the phase contrast technique could improve contrast of subcellular organelles (such as mitochondria, chromosomes, or nuclei), while the DIC images exhibit conspicuous three dimensional profile of cells with embossed visual effect. Furthermore, we performed an extra cell segmentation for counting the cell number, illustrating an approximately linear growth rate over the whole period with the cell number increasing from 121 at 0 h to 449 after 51 h, as shown in Fig. 7(E). All these visualization results and quantitative statistics demonstrate that AIFPM is born for achieving stable QPI and high-resolution morphology analysis of label-free cells with diverse imaging modalities over a long-term period of time.

## Discussion

In this paper, we have reported a video-rate FPM technique based on annular illuminations, named AIFPM, to accomplish high-resolution large-SBP phase retrieval for unstained live samples *in vitro* with the help of 0.4 *NA* annular illuminations and a 10×, 0.4 *NA* objective lens. AIFPM system enables us to improve the resolution and achieve the final effective imaging performance of 0.8 *NA*, corresponding to a full-pitch resolution of 655 *nm* with a FOV of 1.77 *mm*^2^ at a wavelength of 525 *nm*. By reducing the number of required raw images to 4 with a very short exposure time (10 ms), the maximum frame rate of our AIFPM system is enhanced to 25 Hz, providing video-rate label-free quantitative phase imaging videos of living cells. We also investigated the effect of PTF transferring the phase information into intensity in FPM, and it is shown that the use of annular illuminations matching the *NA*_*obj*_ allows for high-accuracy phase reconstruction with a two times super-resolution beyond coherent diffraction limit. Note that this annular illumination strategy is also very useful and has been applied in several TIE based phase retrieval techniques^45,52^. Compared with DPC phase imaging using at least 4 bright-field images with half circle tilted illuminations, AIFPM is able to achieve comparable phase recovery accuracy, higher noise-robustness, and more importantly, provide significant resolution improvement while maintaining a wide FOV, in spite of more time consuming. In summary, the theoretical analysis and experimental results suggest that AIFPM provides new capabilities to raise up the measuring speed and improve low frequency performance of FPM phase imaging, offering a video-rate, high-SBP, label-free means of quantifying biological behavior and dynamic variations over time.

## Electronic supplementary material

Supplementary Information

Supplementary Video 1

Supplementary Video 2

Supplementary Video 3
