# Supplementary material for: High-speed Fourier ptychographic microscopy based on programmable annular illuminations
Source: Sci Rep. 2018 May 16;8:7669. doi: 10.1038/s41598-018-25797-8 (PMC5956106; doi:10.1038/s41598-018-25797-8)
Supplement: Supplementary file 1 — Supplementary Information [file 41598_2018_25797_MOESM1_ESM.pdf]

Supplementary Information:

High-speed Fourier ptychographic microscopy based on programmable annular illuminations

Jiasong Sun[1,2,3], Chao Zuo[1,2,3,*], Jialin Zhang[1,2,3], Yao Fan[1,2,3], and Qian Chen[1,2,*]

[1]School of Electronic and Optical Engineering, Nanjing University of Science and Technology, No. 200 Xiaolingwei Street, Nanjing, Jiangsu Province 210094, China
[2]Jiangsu Key Laboratory of Spectral Imaging & Intelligent Sense, Nanjing, Jiangsu Province 210094, China
[3]Smart Computational Imaging Laboratory (SCILab), Nanjing University of Science and Technology, Nanjing, Jiangsu Province 210094, China
[*]Correspondence and requests for materials should be addressed to C.Z. (email: zuochao@njust.edu.cn) or Q.C. (email: chenqian@njust.edu.cn)

ABSTRACT

This document provides supplementary information for “High-speed Fourier ptychographic microscopy based on programmable annular illuminations”.

Contents:

A. Supplementary Videos

A. Supplementary Videos

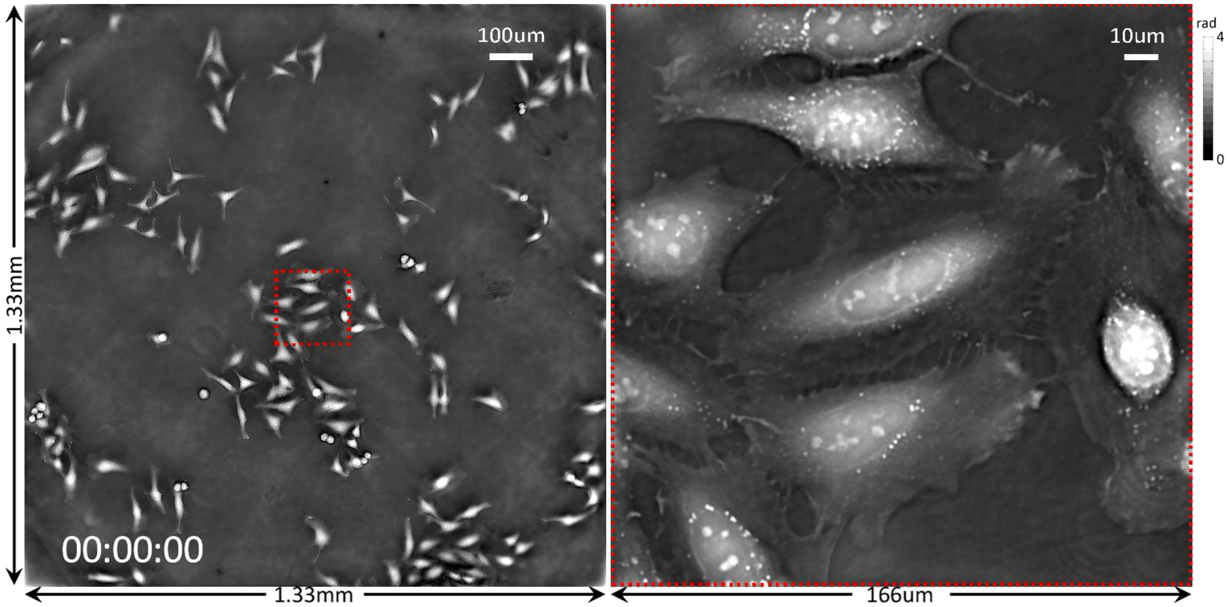

Supplementary Video 1. Large SBP phase video of unstained HeLa cells under-going division over the course of 5 hours at 8.33 Hz frame rate (see also Figure 5).

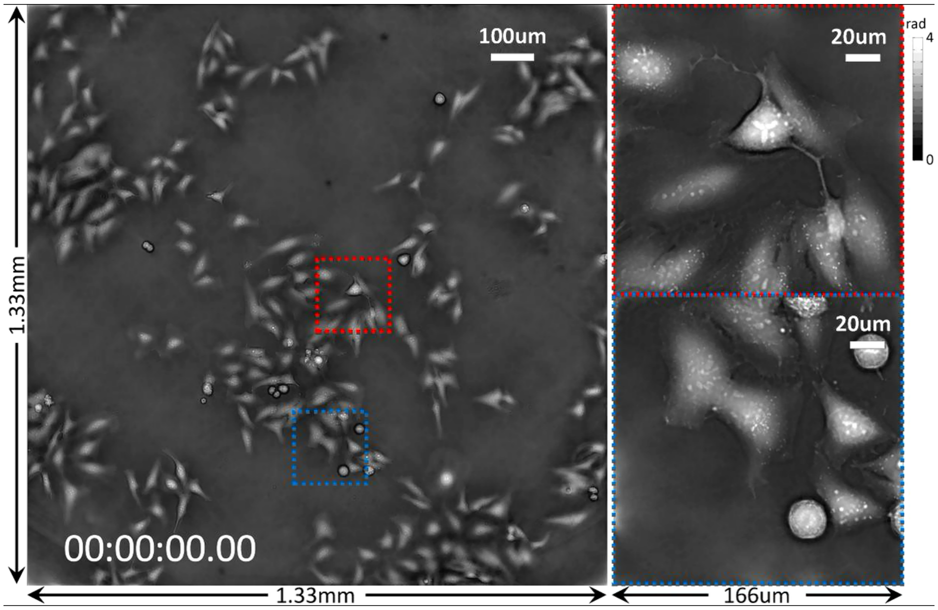

Supplementary Video 2. Large SBP phase video captured with video-rate acquisition speed (25 Hz) for tracking dynamic subcellular features of unstained HeLa cells *in vitro* (see also Figure 6).

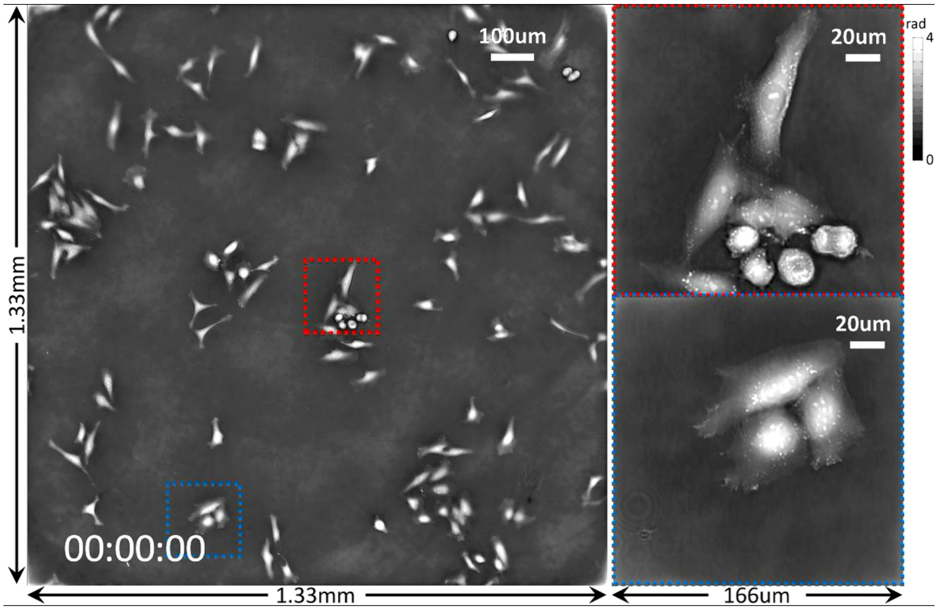

Supplementary Video 3. Long-term large SBP phase video of unstained HeLa cells over a long-term period of 51 hours at 2 minute intervals for analyzing the morphology and statistics quantitatively (see also Figure 7).
